# Bacterial Counts and Physical Properties of Hatching Eggshells Sprayed with a Formaldehyde Solution

**DOI:** 10.3390/antibiotics14100972

**Published:** 2025-09-26

**Authors:** Gabriel da Silva Oliveira, Igor Rafael Ribeiro Vale, Luana Maria de Jesus, Concepta McManus, Heloisa Alves de Figueiredo Sousa, Paula Gabriela da Silva Pires, José Luiz de Paula Rôlo Jivago, Vinícius Machado dos Santos

**Affiliations:** 1 Faculty of Agronomy and Veterinary Medicine, University of Brasília, Brasília 70910-900, Brazil; 2 Laboratory of Poultry Science, Federal Institute of Brasília, Campus Planaltina, Brasília 73380-900, Brazil; 3 Center for Nuclear Energy in Agriculture (CENA), University of São Paulo, São Paulo 13416-000, Brazil; 4 Institute of Biological Sciences, University of Brasília, Brasília 70910-900, Brazil; 5 Laboratory of Microbiology and Food, Federal Institute of Brasília, Campus Planaltina, Brasília 73380-900, Brazil; 6 Animal Production and Health Postgraduate Program, Catarinense Federal Institute, Concórdia 89703-720, Brazil

**Keywords:** eggshell hatching, egg sanitization, eggshell microbiology, poultry, poultry safety, toxic products

## Abstract

Poultry companies must implement measures to sanitize hatching eggs and reduce the risk of bacterial infections associated with poultry management. Many of them use formaldehyde (FA) fumigation in their egg sanitization protocols, but its toxicity has led to recommendations for reducing its use. However, studies employing this approach with liquid FA solutions in poultry operations, particularly during the hatching egg sanitization stage, remain scarce. Our objective was to evaluate whether sprayable FA reduces bacterial contamination on eggshells and whether it causes changes in their physical properties based on the analysis of microstructure, percentage relative to egg weight, and thickness. FA solutions at 0.5, 1, and 2% inhibited bacterial growth in vitro and reduced the bacterial load on the eggshell surface while also causing severe damage to the shell structure. Our results suggest that companies using FA should be aware of the associated risks, as significant production losses may be linked to the shell damage caused by this compound.

## 1. Introduction

Bacteria that contaminate eggs can vary in origin, transmission, severity, and resistance. Harmful strains must be controlled by sanitizing agents to ensure the safety of hatching eggs throughout embryonic development. Studies on poultry health have employed conventional methods to assess the effectiveness of sanitizers in decontaminating hatching eggs, such as measuring residual bacterial levels on eggshells via standard microbiological counts [1,2,3,4,5,6,7,8]. However, relying solely on these traditional approaches does not provide a comprehensive view of the safety and efficacy of sanitizing products such as formaldehyde (FA), especially as they overlook additional factors such as toxicity to poultry. A standardized evaluation of sanitizers that includes microbiological efficacy and eggshell integrity is recommended to support management decisions and improve both productive and sanitary outcomes.

FA is primarily available in its liquid form, known as formalin, due to its high solubility in water and unstable in its pure state [9]. Despite its wide industrial availability, liquid formalin alone has rarely been used in protocols for sanitizing hatching eggs. Instead, it is commonly combined with potassium permanganate to generate FA gas, a method that has proven effective in reducing the bacterial load on the eggshell surface. For example, Amoah et al. [10] reported that sanitizing hatching eggs with 30 mL of 40% FA combined with 20 g of potassium permanganate crystals significantly reduced the bacterial load present on the eggshells. When used in its liquid form, formalin alone can be applied by spraying with manual or automatic sprayers, dispersing a sanitizing mist on the surface of hatching eggs. On the other hand, when combined with potassium permanganate, formalin is placed in a container and subsequently mixed with potassium permanganate, releasing FA gas for egg fumigation in a fully enclosed environment. In addition, FA gas can also be produced by burning paraformaldehyde on a heated plate [11,12]. Typically, the application of FA is carried out after egg collection, and some hatcheries also apply it throughout the incubation and hatching process due to its low residual antibacterial activity [11,13,14,15].

FA is a cytotoxic, genotoxic, teratogenic, and carcinogenic substance, irrespective of its physical state [16,17,18], with limited evidence in the literature regarding its direct toxic effects on eggs intended for incubation. Oliveira et al. [12] reviewed the toxic effects of FA gas on embryos and reported the occurrence of low weight, incomplete development, malformations, and embryonic death. Similarly, liquid FA has been associated with moderate toxicity, as evidenced by the hen’s egg test chorioallantoic membrane assay [19]. Therefore, to the best of our knowledge, there are no reports in the literature on the effects of liquid FA application on the microstructure of eggshells.

To contribute to the consolidation of a more comprehensive database in the scientific literature, which still presents significant gaps regarding the antibacterial and harmful effects of FA in poultry production despite its widespread use in the sector, this study was to evaluate whether sprayable FA reduces bacterial contamination on eggshells and whether it causes changes in their physical properties based on the analysis of microstructure, percentage relative to egg weight, and thickness.

## 2. Results and Discussion

The in vitro disk diffusion assay demonstrated a dose-dependent antibacterial activity profile of the FA sanitizer against *Escherichia coli* (*E*. *coli*) and *Staphylococcus aureus* (*S. aureus*), with inhibition zones ranging from approximately 12 to 20 mm as the formalin concentration increased from 0.5 to 2.0% (Table 1). In agreement with the results of the present study, Mehmood et al. [20] reported inhibition zones of 20.06 and 19.49 mm for *E. coli* and *S. aureus*, respectively, when 0.5% formalin was used. Additionally, Wanja et al. [21] described inhibition zones of 14 and 19 mm for *E. coli* exposed to 1 and 2% formalin, respectively. Even more pronounced results were reported by Fadeyibi et al. [22], who reported inhibition zones of 44.10 mm for *S. aureus* and 28.90 mm for *E. coli* when a formulation containing 40% FA was used. This bacterial inhibition occurs through the reaction of its carbonyl group of FA with amino groups in bacterial proteins and nucleic acids [23].

FA reduced the bacterial load on eggshells, and this reduction depended on both the applied concentration and the post-sanitization evaluation period, with a significant interaction between these factors (*p* < 0.05) (Table 2). The mesophilic bacterial count, recorded at 3.57 ± 0.29 log_10_ CFU/mL after one hour of application, remained unchanged at 3.50 ± 0.17 log_10_ CFU/mL after 72 h (FA0) (*p* > 0.05). All tested FA concentrations reduced the counts after one hour, and they remained lower after 72 h (*p* < 0.05), although a slight trend of bacterial growth was observed over this period. Enterobacteriaceae counts, recorded at 1.20 ± 0.37 log_10_ CFU/mL after one hour, slightly increased to 1.29 ± 0.13 log_10_ CFU/mL after 72 h (FA0) (*p* > 0.05), whereas all FA treatments reduced these counts to <10 CFU/mL after one hour (*p* < 0.05); however, after 72 h, eggshells treated with FA1 and FA2 showed an increase in these counts, reaching levels similar to those of the control (*p* > 0.05), except for the FA3 treatment, which maintained reduced counts after 72 h (*p* < 0.05). The total coliform count, recorded at 1.13 ± 0.16 log_10_ CFU/mL after one hour, decreased to <10 CFU/mL after 72 h (*p* < 0.05). After one hour, FA reduced the total coliform count to <10 CFU/mL regardless of the concentration applied (*p* < 0.05), maintaining this effect after 72 h, which was similar to that of the control (*p* > 0.05). The results obtained in this study corroborate the evidence previously reported in the literature regarding the antibacterial effect of FA. Hasani and Hasani [24] demonstrated that the application of gaseous FA significantly reduced the bacterial load on chicken eggshells, with Enterobacteriaceae counts decreasing from 1.37 to 1.01 log_10_ CFU/egg and mesophilic bacteria populations decreasing from 4.12 to 1.56 log_10_ CFU/egg. Similarly, Vale et al. [25], using a 1.5% aqueous FA solution, reported a marked decline in mesophilic bacteria from 4.96 ± 0.52 to 2.03 ± 0.47 log_10_ CFU/mL and in Enterobacteriaceae from 2.24 ± 1.20 to 1.20 ± 1.31 log_10_ CFU/mL. Consistent with these findings, Al-Shemery and Kamaluddin [26] reported the absence of *Salmonella* spp. and *E. coli* on eggshells following treatment with formalin concentrations similar to those used in this study (0.5, 1, and 1.5%), further reinforcing the efficacy of FA in reducing harmful bacteria regardless of their physical state. Although at 72 h after application, FA3 maintained all the bacterial groups at significantly lower levels than the control, there was a tendency for an increase in the mesophilic bacteria load regardless of the FA3 concentration, as mentioned previously. Furthermore, the results for Enterobacteriaceae counts at FA1 and FA2 highlighted a possible limitation of the residual antibacterial activity of FA.

Reports have compared the antibacterial efficiency of FA with other sanitizers applied to hatching eggs based on the count of mesophilic bacteria on the eggshell. Melo et al. [4] observed that, among the tested compounds (FA, ozone, ultraviolet light, hydrogen peroxide, and peracetic acid), only ultraviolet light and peracetic acid significantly reduced these microorganisms. Clímaco et al. [3] demonstrated that both FA and ultraviolet light decreased this load, with FA being the most effective. This compound showed a performance similar, in both liquid and gaseous forms, to that of essential oils in reducing these microorganisms on the eggshell [25,27]. However, in long-term evaluations, essential oils exhibited greater efficacy, highlighting the limitation of FA in preventing eggshell recontamination [19,28].

**Figure 1:**
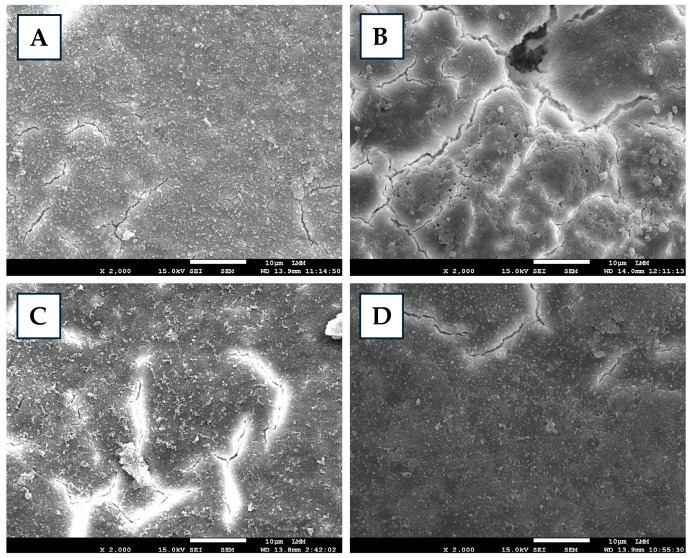
Scanning electron micrographs of eggshells treated or non-treated with formaldehyde (FA) solutions. Control (**A**)—exhibits preserved mineral content and overall shell structure. ((**B**), 0.5%) FA1—presents slight surface smoothing and early signs of mineral loss. ((**C**), 1%) FA2—exhibits increased porosity and fragmented areas. ((**D**), 2%) FA3—presents severe demineralization with a smooth surface, indicating structural weakening.

Different degrees of preservation and structural alterations were observed in eggshells sprayed with varying concentrations of FA (Figure 1). FA0 preserved mineralization, maintaining relative structural stability. FA1 exhibited the onset of mineral loss, accompanied by surface smoothing, indicating progressive but non-critical changes. In FA2, the eggshell, despite maintaining its mineral content, was porous and fragmented, with severe surface irregularities. FA3 presented an advanced stage of degradation, characterized by evident demineralization and a smooth and homogeneous surface, indicating fragilization and structural compromise. Therefore, all concentrations of liquid FA used induced significant damage to the eggshell, which could be erosive or fragmenting, both of which are detrimental to the shell’s strength and protective function. The type and severity of damage were dependent on the concentration of the applied substance. Drastic damage to the eggshell cuticle [29] and compromised eggshell integrity [19] were reported after FA fumigation, corroborating the results observed in the present study. Other sanitizers may also cause damage to the eggshell, such as ozone, peracetic acid, and essential oils [19,30,31]. The latter, when tested in comparison with FA, showed less pronounced effects on the shell [19]. The eggshell structure performs essential functions such as serving as a physical barrier, regulating moisture loss, enabling gas exchange, and protecting against infections [32,33]. Alterations in eggshell integrity caused by FA may impair these processes, creating adverse conditions for the embryo that can ultimately compromise embryonic development and even result in embryonic mortality.

The proportion of eggshell weight relative to egg weight was similar among the treatments (*p* > 0.05) (Table 3). However, FA3 tended toward a lower proportion than the control, a finding that coincides with the evidence of demineralization observed via electron microscopy analysis for the same treatment. Similarly, regarding eggshell thickness, a slight decreasing trend was detected, which was more evident in FA3, although these differences did not reach statistical significance. Clímaco et al. [3] also did not find alterations in eggshell thickness after fumigation with FA, a finding consistent with the absence of differences between eggs sanitized with FA and those treated with sodium chloride, hydrogen peroxide, or propolis [34,35]. Even in the absence of significant variations in macroscopic variables, microstructural analyses reveal substantial alterations in eggshell integrity associated with the application of the FA solution.

## 3. Materials and Methods

The aqueous FA solution used in this study was commercially obtained, containing 36.5–38% FA and a density of 1.08 kg/L. To evaluate the antibacterial efficacy of FA using the disc diffusion method [36], bacterial strains of *E. coli* and *S. aureus* (American Type Culture Collection, Manassas, VA, USA) were grown in brain heart infusion broth and incubated for 24 h at 36 °C. After incubation, each bacterial inoculum was standardized to a turbidity equivalent to 0.5 on the McFarland scale, and 0.1 mL was spread onto the surface of Petri dishes containing Mueller‒Hinton agar. Sterile discs impregnated with FA solutions at concentrations of 0.5, 1, and 2% were placed on the agar surface in triplicate. These concentrations were selected based on the studies of Al-Shemery and Kamaluddin [26] and Wanja et al. [21]. Discs containing distilled water were used as negative controls. The plates were incubated for 24 h at 36 °C, and the average diameter of the inhibition zones in triplicate was recorded.

The eggs used in this study were non-hatching brown eggs, as the focus was exclusively on the external structure. These eggs were treated with FA at concentrations of 0.5, 1, and 2% (designated as treatments FA1, FA2, and FA3, respectively), diluted in distilled water, and, after drying for one hour, subjected to scanning electron microscopy, analysis of eggshell percentage and thickness, or bacteriological analysis, with the latter repeated after 72 h. During this period, the eggs were stored at a temperature of 24 ± 2 °C. Spraying was performed manually, egg by egg, using a hand sprayer in a room specifically designated for egg sanitization, with the use of personal protective equipment. Non-sanitized eggs were used as the negative control (FA0).

For microstructure analysis, sanitized eggshell samples were prepared in triplicate for each tested concentration [37], subjected to metallization and examined using a JEOL JSM-7001F scanning electron microscope (Jeol Ltd., Akishima, Tokyo, Japan) at a standard magnification of up to ×4000 [19]. The images were analyzed, and morphological, textural, and structural alterations were recorded. Eggshell percentage was calculated based on egg weight, and eggshell thickness, including that of the membrane, was measured using a precision digital caliper (Mitutoyo, São Paulo, Brazil), with averages obtained from three different points along the equatorial plane of the shell.

For the bacteriological analysis, 0.1 mL of the solution obtained from washing each eggshell in a sterile plastic bag containing 0.1% peptone saline solution (five repetitions for each tested concentration) and its serial dilutions were plated on plate count agar (Ionlab, Paraná, Brazil), violet red bile glucose agar (Ionlab, Paraná, Brazil), and violet red bile lactose agar (Kasvi, Paraná, Brazil) for the enumeration of total aerobic mesophilic bacteria, Enterobacteriaceae, and total coliforms, respectively, after incubation at 36 °C for 48 h. The average number of colonies was recorded. For the FA0 samples, the same procedure was followed.

The data were checked for normality using PROC UNIVARIATE in SAS Studio 9.4 University Edition software (SAS Inst. Inc., Cary, NC, USA). Non-parametric data were analyzed via the Kruskal‒Wallis test from PROC NPAR1WAY. PROC GLM was used for analysis of variance on the data. Significant differences were reported as *p* < 0.05 according to Tukey’s test.

## 4. Conclusions

Sanitization of hatching eggs is a method used to prevent bacterial dissemination in poultry farms. This process is increasingly integrated into poultry sanitary management, and its use should not be overlooked. However, in line with existing knowledge on FA fumigation for egg sanitization, we have shown that spraying with a liquid FA solution is also effective in controlling bacterial contamination on the eggshell surface but may compromise its integrity, and consequently, its function. This set of eggshell evaluations represents a novel contribution to the research field, highlighting the risks associated with the use of sprayable FA, already applied in some hatcheries but still poorly described. To date, no studies have analyzed egg spray protocols with sprayable FA, as tested in this work, in association with the set of analyses of eggshell physical properties, which possibly makes this the first study to explore this relationship in more detail. The results expand current knowledge by demonstrating that the liquid spray application of FA is not a safe alternative to gaseous fumigation in terms of its impact on eggshell structural properties, generally reinforcing the need to consider alternatives not only to prevent severe shell damage but also to ensure good antibacterial efficacy and greater safety for the embryo, such as hydrogen peroxide and essential oils.

## Figures and Tables

**Table 1:** Antibacterial profile of formaldehyde (FA) assessed by the disk diffusion test.

Bacterial Strains	Concentrations (%)			
	0.0 (Negative Control)	0.5	1.0	2.0
	Mean Inhibition Halo (mm) ± Standard Deviation			
*E. coli*	0.00	12.25 ± 0.40	13.70 ± 0.27	17.12 ± 0.79
*S. aureus*	0.00	14.92 ± 0.36	16.89 ± 0.12	20.18 ± 0.87

**Table 2:** Bacterial counts on eggshells treated or non-treated with formaldehyde (FA) solutions.

FA Treatment	Time After Spraying (h)	
	1	72
	Mesophilic Bacterial Count (log_10_ CFU/mL)	
FA0	3.57 ± 0.29 ^A,a^	3.50 ± 0.17 ^A,a^
FA1	1.58 ± 0.10 ^B,b^	2.15 ± 0.43 ^A,b^
FA2	1.44 ± 0.25 ^B,b^	2.23 ± 0.17 ^A,b^
FA3	1.47 ± 0.20 ^B,b^	2.10 ± 0.25 ^A,b^
*p* value		
T	<0.0001	
P	<0.0001	
T × P	0.0021	
**FA Treatment**	**Time After Spraying (h)**	
	**1**	**72**
	**Enterobacteriaceae (log_10_ CFU/mL)**	
FA0	1.20 ± 0.37 ^A,a^	1.29 ± 0.13 ^A,a^
FA1	<10 CFU/mL ^B,b^	1.11 ± 0.23 ^A,a^
FA2	<10 CFU/mL ^B,b^	1.00 ± 0.19 ^A,a^
FA3	<10 CFU/mL ^A,b^	<10 CFU/mL ^A,b^
*p* value		
T	<0.0001	
P	<0.0001	
T × P	<0.0001	
**FA Treatment**	**Time After Spraying (h)**	
	**1**	**72**
	**Total Coliform Count (log_10_ CFU/mL)**	
FA0	1.13 ± 0.16 ^A,a^	<10 CFU/mL ^B,a^
FA1	<10 CFU/mL ^A,b^	<10 CFU/mL ^A,a^
FA2	<10 CFU/mL ^A,b^	<10 CFU/mL ^A,a^
FA3	<10 CFU/mL ^A,b^	<10 CFU/mL ^A,a^
*p* value		
T	<0.0001	
P	<0.0001	
T × P	<0.0001	

^a, b, A, B^ Different lowercase letters in the same column and uppercase letters in the same row indicate significant differences according to Tukey’s test (*p* < 0.05). Formaldehyde spraying at 0.5% (FA1), 1% (FA2), or 2% (FA3). T, Treatment; P, Period.

**Table 3:** Macroscopic analysis of eggshells treated or non-treated with formaldehyde (FA) solutions.

Formaldehyde Treatment	Egg Weight (g)	Eggshell Percentage (%)	Eggshell Thickness (mm)
FA0	56.92 ± 0.56 ^a^	10.14 ± 0.51 ^a^	0.351 ± 0.01 ^a^
FA1	56.80 ± 0.72 ^a^	9.91 ± 0.48 ^a^	0.349 ± 0.01 ^a^
FA2	56.72 ± 0.64 ^a^	9.95 ± 0.60 ^a^	0.347 ± 0.01 ^a^
FA3	56.60 ± 0.64 ^a^	9.79 ± 0.44 ^a^	0.341 ± 0.02 ^a^

^a^ Same letters in the same column indicate no significant differences according to Tukey’s test (*p* > 0.05).

## Data Availability

The data are contained within the article.
